# Prevalence of H63D, S65C and C282Y hereditary hemochromatosis gene mutations in Slovenian population by an improved high-throughput genotyping assay

**DOI:** 10.1186/1471-2350-8-69

**Published:** 2007-11-23

**Authors:** Marko Cukjati, Tomaž Vaupotič, Ruth Rupreht, Vladka Čurin-Šerbec

**Affiliations:** 1 Blood Transfusion Centre of Slovenia, Šlajmerjeva 6, 1000 Ljubljana, Slovenia; 2 Institute of Biochemistry, University of Ljubljana, Medical Faculty, Vrazov trg 2, 1000 Ljubljana, Slovenia

## Abstract

**Background:**

Hereditary hemochromatosis (HH) is a common genetic disease characterized by excessive iron overload that leads to multi-organ failure. Although the most prevalent genotype in HH is homozygosity for C282Y mutation of the *HFE *gene, two additional mutations, H63D and S65C, appear to be associated with a milder form of HH. The aim of this study was to develop a high-throughput assay for *HFE *mutations screening based on TaqMan technology and to determine the frequencies of *HFE *mutations in the Slovenian population.

**Methods:**

Altogether, 1282 randomly selected blood donors from different Slovenian regions and 21 HH patients were analyzed for the presence of *HFE *mutations by an in-house developed real-time PCR assay based on TaqMan technology using shorter non-interfering fluorescent single nucleotide polymorphism (SNP)-specific MGB probes. The assay was validated by RFLP analysis and DNA sequencing.

**Results:**

The genotyping assay of the H63D, S65C and C282Y mutations in the *HFE *gene, based on TaqMan technology proved to be fast, reliable, with a high-throughput capability and 100% concordant with genotypes obtained by RFLP and DNA sequencing. The observed frequency of C282Y homozygotes in the group of HH patients was only 48%, others were of the heterogeneous *HFE *genotype. Among 1282 blood donors tested, the observed H63D, S65C and C282Y allele frequency were 12.8% (95% confidence interval (CI) 11.5 – 14.2%), 1.8% (95% CI 1.4 – 2.5%) and 3.6% (95% CI 3.0 – 4.5%), respectively. Approximately 33% of the tested subjects had at least one of the three HH mutations, and 1% of them were C282Y homozygotes or compound heterozygotes C282Y/H63D or C282Y/S65C, presenting an increased risk for iron overload disease. A significant variation in H63D allele frequency was observed for one of the Slovenian regions.

**Conclusion:**

The improved real-time PCR assay for H63D, S65C and C282Y mutations detection is accurate, fast, cost-efficient and ready for routine screening and diagnostic procedures. The genotype frequencies in the Slovenian population agree with those reported for the Central European populations although some deviations where observed in comparison with other populations of Slavic origin. Regional distribution of the mutations should be considered when planning population screening.

## Background

Hereditary hemochromatosis (HH) is an inherited autosomal recessive disorder of iron metabolism. Due to excessive intestinal absorption, iron accumulates in parenchymal cells of the liver, pancreas, heart and other organs with resultant damage to their structure and impairment of their function. It is one of the most common genetic diseases in Caucasians with a prevalence of nearly 1 in 300 [1]. Although the symptoms of the disease are often nonspecific, much of the organ damage is irreversible once it has occurred. Early detection and therapy is therefore very important as a part of preventive medicine. The discovery of the responsible gene *HFE *in 1996 enabled molecular analysis to be included in the diagnostic strategy for HH [2]. A number of different *HFE *mutations have been reported so far. The majority of HH cases (52–96%) in European regions are associated with a homozygous 845G→A mutation within exon 4 of the *HFE *gene, which results in amino acid change at position 282 from cysteine to tyrosine (C282Y) [1,2]. A second mutant allele 187C→G detected with relatively high frequency occurs within the exon 2 of the *HFE *gene where aspartate replaces histidine at amino acid position 63 (H63D) [2]. The contribution of this allele to iron overload is most relevant in the case of combined heterozygosity with C282Y allele (C282Y/H63D) [3,4]. The third common mutation of *HFE *is 193A→T substitution in exon 2 (S65C) and was shown to be generally benign, although a C282Y/S65C genotype may confer a slight increase in disease risk, contributing to a mild disease phenotype [4-6]. Many of the other *HFE *mutations described are private and rare, or they were found to be significantly enriched only in certain regions [7]. Therefore the frequencies of the *HFE *mutations in the general population and in patients with clinically expressed disease have to be established in order to implement appropriate genetic tests in diagnostic and screening procedures for HH. There are very few studies providing information about the frequency of any of these three major HH mutations in populations of Slavic origin [8-12]. Only one study, based on RFLP method, is reported for the Slovenian population and is based on a limited number of subjects [11]. However, we have no data yet about the frequency of the *HFE *mutations in the Slovenian healthy population and in patients with clinically expressed disease nor do we have data about distribution of HH genotypes in different Slovenian regions.

Since HH is a genetic disorder that fulfils most of the WHO criteria for large-scale population screening program [13], the availability of a cost-effective method is an important issue. Although genetic testing for HH is generally not recommended as the first step in a wide population screening program because of uncertainty about the natural history of the disease, age-related penetrance and the psychosocial impact of genetic testing, its major role in confirmation of the diagnosis cannot be overlooked. Furthermore, family genetic testing performed among the relatives of a newly diagnosed patient also enables detection of subjects in the pre-symptomatic phase [14,15].

Many PCR-based methods were reported for HH mutation detection, however, real-time PCR-based assays with TaqMan technology seems to be one of the most prominent among them. The fluorescent TaqMan probes with minor groove binder (MGB) are shorter and thus significantly more specific for single nucleotide polymorphisms (SNP) detection than standard DNA probes or allele specific PCR derivatives. The technology itself is completely flexible and suitable for the extremely high-throughout screening procedures since it combines the PCR amplification and detection into a single step therefore also minimizing the possibility of contamination [16]. An objection of so far reported TaqMan-based assays for the H63D and C282Y mutation detection was mainly the improper design of the probes for *HFE *codon 63 screening, which are too long and overlap the polymorphic position of the *HFE *codon 65 [16-18]. This could lead to the misinterpretation of results obtained by those probes and therefore more optimized probes are critical for a reliable genotyping assay. It is worth to stress that the TaqMan probes for the detection of the S65C polymorphism have not been reported to date.

The aim of our work was (i) to develop an accurate, rapid and cost-effective diagnostic assay based on the TaqMan technology for the high-throughput detection of 187C→G, 193A→T and 845G→A mutations in the *HFE *gene with validation on a small group of HH patients and (ii) to determine the frequencies of *HFE *mutations in the healthy population in Slovenia and to compare them with some other European populations, especially those of Slavic origin. The method described in this paper is now available for routine diagnostic and screening procedures for HH, opening new possibilities in preventive medicine and possibly in screening for potential new blood donors.

## Methods

### Patients and control subjects

Voluntary, unpaid, unrelated whole blood donors who attended blood donor sessions in 11 geographic regions of the Republic of Slovenia from May 2002 to January 2003 were randomly selected for the population study. Blood samples were obtained from 1282 blood donors, 934 male and 348 female, aged between 18 and 64 years (40.4 ± 10.0 years for male and 39.7 ± 9.6 years for female). In addition, 21 patients, 20 men and 1 woman, with clinical and biochemical characteristics of HH who underwent therapeutic phlebotomy were included in the study. The mean age was 53 years for male patients (25 to 73 years) and 36 for female patient. In all patients the diagnosis of HH was confirmed by quantitative phlebotomy with removal of more than 4 grams of iron. The study was approved by the National medical ethics committee number 66/05/2002 and informed consent was obtained from all volunteers.

### Genomic DNA isolation

Genomic DNA was isolated from 300 μL of buffy coat using QIAamp Blood kit (Qiagen) according to the manufacturer's instructions (Qiagen).

### Real-time PCR

Primers and TaqMan^® ^MGB probes were designed using Primer Express v2.0 software (Applied Biosystem) based on the published sequence of the *HFE *gene (GeneBank ID NM000410). We carefully designed the probes for codons H63D and S65C (SNPs being only six nucleotides apart) so that they do not overlap. PCRs for each SNP were performed in a single reaction tube for wild type and mutant allele simultaneously on thermostable 96-well plate in ABI PRISM 7900HT Sequence Detection System (Applied Biosystems). A 20 μL reaction consisted of TaqMan Universal PCR Master Mix with the passive reference ROX (Perkin Elmer), 50 ng of genomic DNA, 300 nM of each primers 5'-TTGGGCTACGTGGATGACC-3' and 5'-TCTGGCTTGAAATTCTACTGGAAA-3' for mutation H63D and S65C or 5'-GAACCTAAAGACGTATTGCCAA-3' and 5'-AGATCACAATGAGGGGCTGATC-3' for mutation C282Y and a corresponding pair of TaqMan MGB probes: 75 nM of each 5'-FAM-CTCATCATCATAGAACAC-NFQ-3' (mutated) and 5'-VIC-CTCATGATCATAGAACAC-NFQ-3' (wild-type) for H63D, 70 nM 5'-VIC-ACGGCGACACTCA-NFQ-3' (mutated) and 90 nM 5'-FAM-CGGCGACTCTCA-NFQ-3' (wild-type) for S65C or 45 nM 5'-VIC-CCTGGTACGTATATCT-NFQ-3' (mutated) and 55 nM 5'-FAM-CTGGCACGTATATCT-NFQ-3' (wild-type) for C282Y allele detection. PCR conditions were 2 min at 50°C, 10 min at 95°C, followed by 40 cycles of 15 sec at 95°C and 1 min at 60°C carried out by the ABI PRISM 7900HT Sequence Detection System (Applied Biosystem). Allele discrimination was accomplished by running end point detection using ABI PRISM 7900HT and SDS 2.0 software. In addition, all results were confirmed by visual inspection of the real-time PCR multicomponent analysis plots.

### RFLP analysis

Exons 2 and 4 of *HFE *gene were separately amplified by PCR prior to the restriction analysis and sequencing in a 50 μL reactions consisted of 200 nM of each primer 5'-TGTGGAGCCTCAACATCCT-3 and 5'-TGAAAAGCTCTGACAACCTCA-3' for exon 2 or 5'-TCCAGTCTTCCTGGCAA-3' and 5'-TTCTAGCTCCTGGCTCTCA-3' for exon 4, 10× PCR buffer (Perkin Elmer), 200 μM each dNTP (Perkin Elmer), 1.25 mM MgCl_2 _(Perkin Elmer), 3 U *Taq *DNA polymerase (Perkin Elmer) and 200 ng genomic DNA. The thermal program consisted of 30 cycles with 20 sec at 94°C, 30 sec at 56°C and 1 min at 72°C. After PCR amplification, restriction digests were performed directly with the 25 μL of PCR mixtures by the addition of 5U *Bcl*I (codon 63), *Hinf*I (codon 65) or *SnaB*I (codon 282) and corresponding buffers and incubated for 2 h at 50° for *Bcl*I restriction or at 37°C for *Hinf*I and *SnaB*I restriction. The products were resolved on a 3% agarose gel. Genotypes of HH patients were also confirmed by DNA sequencing (Macrogen Inc., Seoul, Korea).

### Statistical analysis

The results are expressed as means ± SD. Allele frequencies are presented as % with 95% confidence interval (95% CI) calculated by the Wilson procedure with a correction for continuity. Fisher's exact test or χ^2 ^test with Yates correction was used to compare the prevalence of *HFE *genotypes among different groups by age and gender and to compare allelic frequencies among different European populations. The fit to the Hardy-Weinberg (H-W) equlibrium was tested by means of the χ^2 ^test. Data analysis was carried out using the statistical package SigmaStat 3.1 (Systat software Inc., San Jose, CA).

## Results

### The improved TaqMan MGB probes simultaneously and accurately discriminate 187C/G, 193A/T and 845G/A positions in the *HFE *gene

**Figure 1:**
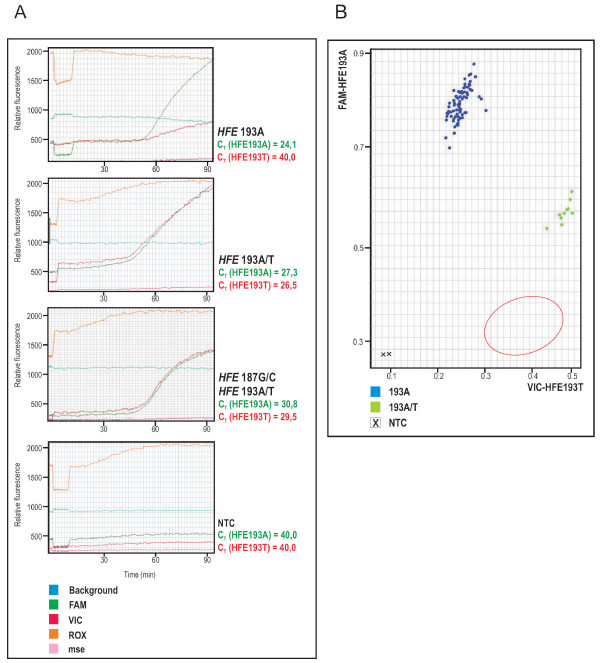
**Validation of the real-time PCR assay for the 193A→T (S65C) mutation detection**. (A) A multicomponent real-time amplification plots created by the SDS software. The genotypes are indicated with bold. The C_T _values of individual probes for all genotypes are presented. Curves in the plots correspond to the indicated fluorophores or water (see the legend); ROX, internal reference dye; mse, mean squared error; NTC, no-template control. (B) End-point fluorescence detection is shown by the scattered diagram. Clustering of genotypes is based on the relative fluorescence from each well on 96-well plate. The expected area for 193 mutant homozygote samples clustering is indicated by red circle.

The universal cycling program of the real-time PCR was performed at the same temperature conditions for all probes designed in the study. The genotyping results obtained by the real-time PCR analysis using the TaqMan MGB probes are graphically presented for the case of S65C mutation detection in Figure 1. The fluorescence of only one of the two probes in the reaction mixture increased exponentially in the case of the homozygous genotype, either wild-type or mutant (Fig. 1A, upper panel represents 193A homozygous genotype), whereas a comparable and simultaneous increase in fluorescence of both probes at similar C_T _values was observed in the case of the heterozygous genotype (Fig. 1A, second panel represents 193A/T heterozygous genotype). The reliability of the codon 65 genotyping using HFE193A and HFE193T probes in the presence of the polymorphisms at both positions, 187 and 193, is shown in the case of the H63D/S65C combined heterozygote. As shown in Fig. 1A, third panel, the SNP at position 193 was successfully and accurately identified without any interfering effects from the SNP at position 187, which was not true for the probe that covers both polymorphic positions at the same time (data not shown). The scattered diagram showing only relative end-point fluorescence intensities of both TaqMan MGB probes for allelic discrimination of the *HFE *codon 65, is shown in Figure 1B. Clustering of wild type homozygous and heterozygous samples within the 96-well plate was unambiguously evident from the end-point fluorescence measurements, which makes allelic discrimination even more routine and faster.

### Validation of the real-time PCR assay by the RFLP analysis and DNA sequencing

Altogether, 21 HH patients were included for validation of the real-time PCR. Only 10 of them (48%) were found to be homozygous for C282Y mutation. Two patients were homozygotes for H63D mutation. All the others were of the heterogeneous genotypes: one patient was C282Y heterozygote and two were H63D heterozygotes. Among combined heterozygotes, two were H63D/C282Y and two were H63D/S65C. Two HH patients (10%) lacked any of the three mutations studied here. Positive results were further confirmed by the RFLP analysis and DNA sequencing. No discrepancies were observed between the genotypes obtained by any of three methods. Besides the confirmation of the three mutations in samples by two independent methods, we have gained standards for future testing.

### Frequencies of the *HFE *gene mutation – H63D, S65C and C282Y – in Slovenian blood donors

Genotype frequencies were analyzed in the population of Slovenian blood donors (Table 1). Among 1282 blood donors tested, 30 (2.3%) were H63D homozygotes and 268 (20.9%) were H63D heterozygotes. Two (0.16%) homozygotes and 89 (6.9%) heterozygotes for C282Y mutation were found (Table 1). 47 samples (3.7%) were S65C heterozygotes and no sample was identified as S65C homozygote. Two, six and three samples were H63D/S65C, H63D/C282Y and S65C/C282Y compound heterozygotes, respectively. The calculated H63D, S65C and C282Y allele frequencies were 12.8% (95% confidence interval (CI) 11.5 – 14.2%), 1.8% (95% CI 1.4 – 2.5%) and 3.6% (95% CI 3.0 – 4.5%), respectively (Table 2). There were no significant deviations from the H-W equilibrium in the blood donor population sample and no difference in genotype frequency among blood donor groups differing in gender or age.

**Table 1:** Genotype frequencies of H63D, S65C and C282Y mutations in *HFE *gene in Slovenian blood donors.

**Genotype**			**Frequency**	
**H63D**	**S65C**	**C282Y**	**n**	**% (95% CI)**
-/-	-/-	-/-	857	66.8 (64.2–69.4)
+/-	-/-	-/-	260	20.3 (18.1–22.6)
-/-	-/-	+/-	80	6.2 (5.0–7.7)
-/-	-/+	-/-	42	3.3 (2.4–4.5)
+/+	-/-	-/-	30	2.3 (1.6–3.4)
-/-	-/-	+/+	2	0.16 (0.03–0.63)
-/-	+/+	-/-	0	-
+/-	-/-	+/-	6	0.47 (0.19–1.07)
+/-	+/-	-/-	2	0.16 (0.03–0.63)
-/-	+/-	+/-	3	0.23 (0.06–0.74)
				
***Total***			**1282**	

The presence or absence of the indicated mutation is shown by + or -, respectively.

**Table 2:** Allele frequencies of H63D, S65C and C282Y mutation in the *HFE *gene in Slovenian blood donors.

**HFE alleles**	**Allele frequency % (95% CI)**
**H63D**	12.8 (11.5 – 14.2)
**S65C**	1.8 (1.4 – 2.5)
**C282Y**	3.6 (3.0 – 4.5)

**Figure 2:**
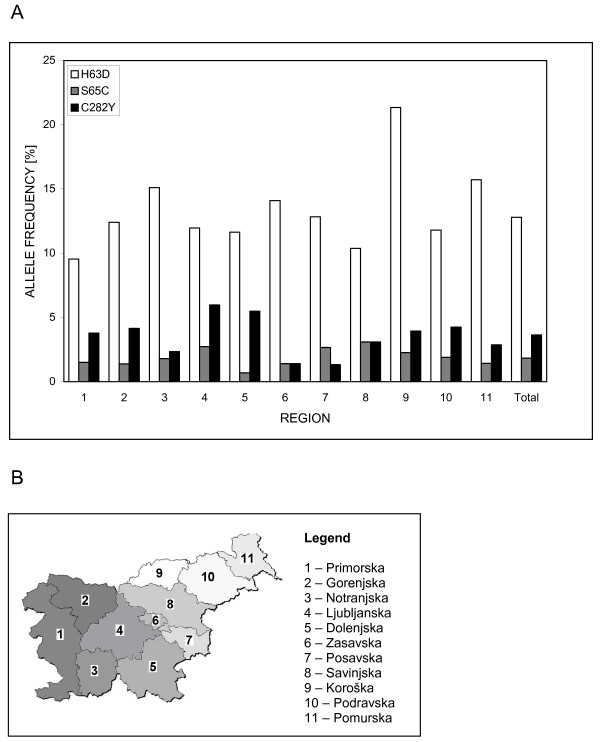
**H63D, S65C and C282Y allele frequency distribution in 1282 blood donors among different Slovenian regions**. A) Regional distribution of *HFE *polymorphisms. The regions, numbered from 1 to 11, are arranged from west to east, and marked on the map below. Average frequency for Slovenia is presented in the last column (total). In the region 9 H63D allele frequency is significant higher comparing to other regions (p = 0.005). (B) Geographic regions of Slovenia.

Comparison of allele frequencies between different Slovenian regions revealed a significantly higher value for H63D allele in Koroška (region 9) in the northeastern part of Slovenia (21.3 % vs average 12.8 %; p = 0.005). Other two alleles were more evenly distributed throughout Slovenia (Figure 2).

## Discussion

### The improved TaqMan MGB probes were optimized for high-throughput genotyping capability

We have optimized an accurate, rapid and high-throughput method for HH mutation detection based on TaqMan technology. Our contribution to the existing TaqMan-based fluorescent probes for *HFE *mutations detection was especially the construction of short specific MGB probes for SNP detection at positions 187 and 193. Using the ABI PRISM 7900HT Sequence Detection System and SDS software, identification of samples with H63D, S65C and C282Y mutations in the *HFE *gene was simple and highly accurate. As shown in Figure 1, the real-time PCR results were easy to interpret either from the multicomponent amplification plot (Fig. 1A) or from the scattered diagram of the end-point fluorescence measurements (Fig. 1B). Genotype calls, as determined by real-time PCR analysis, were 100% concordant with genotypes obtained by RFLP and DNA sequencing.

We have also avoided the irregularity of many so far reported probes for analysis of the *HFE *position 187 by carefully designed short MGB probes for independent determination of both 187 and 193 genotypes. Even though the SNPs being only 6 nucleotydes apart the probes do not overlap the positions 187 and 193 and therefore do not interfere in the assay. Therefore, we have improved accuracy of the H63D genotyping assay. To date, our study is also the first time report of the population based S65C polymorphism genotyping in the *HFE *gene with the use of fluorescent TaqMan MGB probes with a nonfluorescent quencher in a high-throughput manner. Although we have improved real-time PCR assay for H63D, S65C and C282Y mutations detection, much larger surveys with *HFE *genotyping using several assay systems should not be overlooked [19-21].

### *HFE *polymorphism for C282Y was underrepresented in the group of Slovenian HH patients

A small group of Slovenian patients with a clinically expressed disease who underwent therapeutic phlebotomy was included into our study to get positive controls for the test validation as well as for later standard preparation. Although the number of subjects is too small to make any serious conclusion it is evident from our results that the frequency of C282Y homozygotes in the group of HH patients is low (only 48%) when compared with other European populations (52–96%) [1]. In 10% of HH patients none of the three major mutations were found. After further studies, including DNA sequencing, other mutations elsewhere in the *HFE *gene, such as frame-shift deletion c.471del in exon 3 resulting in a premature termination of a nonsense HFE protein, were recently found in one of our HH patients [22]. Mutations in *ferroportin1 *or *TFR2 *genes might also be involved with the phenotypic expression of HH symptoms in these patients and additional studies are on the way.

### H63D allele frequencies differ among different Slovenian regions

To demonstrate the high-throughput capability of the assay, we have performed an investigation on 1282 blood donors from the Slovenian population as healthy representatives by analyzing the occurrence of H63D, S65C and C282Y single nucleotide polymorphisms within the coding region of the *HFE *gene. Since Slovenia has only 2 million residents, the regionally representative group of blood donors presents a very large sample. Allele frequencies of H63D, S65C and C282Y mutations were 12.8%, 1.8% and 3.6%, respectively (Table 2). Based on our results, homozygosity for the C282Y mutation was estimated to occur in 0.16% of the selected population which corresponds to 1 in 641 individuals. Compound heterozygosity for C282Y/H63D occurred in approximately 0.5% of probands. Taken together, approximately 1% of the Slovenian population are C282Y homozygotes or compound heterozygotes C282Y/H63D or C282Y/S65C, which all represent an increased risk for iron overload. More than 33% of tested subjects had at least one of the three mutations, linked to HH.

Comparing allele frequencies among different Slovenian regions revealed significantly higher frequency of H63D allele in one of northeastern regions (Fig. 2). The difference could be explained by the fact that the observed region is surrounded by mountains, characterized by relatively low migratory fluxes, and therefore a relatively isolated geographic area. Although the sample size for this region is too small to check for H-W equilibrium, the frequencies of H63D genotypes are within expected values. Regional distribution of H63D allele should be taken into account when planning wide population screening.

### *HFE *polymorphism prevalence in Slovenian population differs from those of other Slavic populations

In Europe, the population movements have contributed to the ethnic groups, cultures, and consequently, inheritance mixing. HH is one of the genetic diseases whose distribution is directly related to the population movements. The C282Y allele frequencies in the general population are distributed among a decreasing cline from northwest to southeast Europe with a higher prevalence in the populations having a heavier Celtic or Viking component [23-25]. In Table 3, the frequency of the H63D, S65C and C282Y mutations observed in the Slovenian population were compared with the frequencies observed in some other European populations. The prevalence of C282Y mutation in the Republic of Slovenia (3.6%) correlates with the reported northwest to southeast gradient [1] and geographic position of Slovenia. Compared to other nations of Slavic origin the frequency of the C282Y mutation is higher, but not significantly, comparing to Poland (3.1%) [10], the Czech Republic (3.4%) [8] and Croatia (3.3%) [11], and lower than in Bosnia and Herzegovina (4.0%) [12].

Interestengly, the C282Y frequency significantly differs from the population of Serbia and Montenegro (1.6%) [26], also Slavic in origin. Obviously, there is a considerably high difference in C282Y allele frequencies among nations of Slavic origin, which could support a late introduction of the mutation in Slavic nations or, alternatively, that more time passed, than generally believed, from the close connections among Slavic nations. In contrast, the frequency of C282Y mutation in Slovenia is considerably higher than in the Modena region of Italy [27].

**Table 3:** Comparison of allele frequencies of H63D, S65C and C282Y in the Slovenian population with other European populations

**Country**	**n**	**H63D (%)**	**p value**	**S65C (%)**	**p value**	**C282Y (%)**	**p value**
Slovenia*	1282	**12.8**		**1.8**		**3.6**	
Croatia* [11]	200	**14.5**	NS	**1.8**	NS	**3.3**	NS
Hungary [30]	240	**14.4**	NS	**-**	-	**3.4**	NS^a^
Italy – Modena [27]	606	**14.9**	NS	**0.7**	0.015	**4.7**	NS
Italy – Ossola [29]	2100	**14.2**	NS	**-**	-	**1.4**	< 0.001
Bosnia and Herzegovina* [12]	200	**11.3**	NS	**-**	-	**4**	NS
Serbia and Montenegro* [26]	318	**15.7**	NS	**1.6**	NS	**1.6**	0.012
The Czech Republic* [9]	481	**15**	NS	**1.2**	NS	**3.4**	NS
Poland* [10]	871	**16.2**	0.002	**-**	-	**3.1**	NS
Germany [31]	500	**10.8**	NS	**-**	-	**4.5**	NS
Denmark [32]	2501	**13.3**	NS	**-**	-	**5.7**	< 0.001
France [4]	410	**14**	NS	**1.95**	NS	**7.7**	< 0.001
United Kingdom [21]	10556	**15.3**	< 0,001	**-**	-	**8,2**	< 0.001

n, number of subjects examined; -, no data; NS not significant. The significance level (α) was set at 0.05.

^a ^Number of examined subjects was 996.

*Population of Slavic origin

H63D mutation is more frequent and more uniformly distributed among European nations [1]. The highest frequencies of the H63D mutation among Basques and Catalans in Spain suggest that this mutation may have its origin in the early West European populations before the Indo-European expansion [28]. Considering the allele frequency of H63D mutation (12.8%), Slovenia has lower frequency when compared with Poland (16.2%), the Czech Republic (15.0%), Croatia (14.5%) and Serbia and Montenegro (15.7%). Among nations of Slavic origin only Bosnia and Herzegovina was reported to have lower H63D frequency (11.3 %) although the difference is not significant.

The allele frequency of S65C mutation in the Slovenian population was estimated to be 1.8%, which is very close to Croatia (1.8%) and Serbia and Montenegro (1.6%). Interestingly, the S65C allele frequency is also close to the French population in Brittany (1.95%) [4] and higher if compared with the Ossola region in Northern Italy (0.7%) [29]. For the S65C mutation, the population data are so far too scanty to allow for tracing its origin.

## Conclusion

In conclusion, an improved TaqMan high-throughput validated assay, described in this paper is ready for wider screening application. The observed genotype frequency for H63D, S65C and C282Y mutations in the Slovenian population agrees with those reported for the Central European populations, although some deviations were observed in comparison with populations of Slavic origin. A revision of restrictions by the national guidelines on blood donation from otherwise healthy HH patients should be done before the implementation of population screening for HH in order to promote more blood donors necessary to mantain adequate blood supply.

## Competing interests

The author(s) declare that they have no competing interests.

## Authors' contributions

MC and TV contributed equally to this study. MC studied the population, supervised the region-based blood collecting procedure, participated in HH patients recruiting, performed statistical data analysis, and co-drafted the manuscript. TV isolated DNA, designed primers and probes, performed real-time PCR and RFLP genotyping, analyzed results and co-drafted the manuscript. RR supervised the genotyping, designed primers and probes, participated in the study design and results verification. VČŠ conceived the study, participated in its design, coordination and managing, and co-drafted the manuscript. All authors read and approved the final manuscript.

## Pre-publication history

The pre-publication history for this paper can be accessed here:


